# Luxatio Erecta Humeri After a Ground-Level Fall in an Elderly Woman With a History of Ipsilateral Rotator Cuff Repair: A Case Report

**DOI:** 10.7759/cureus.27650

**Published:** 2022-08-03

**Authors:** Shayan A Memar, Jake Stahnke, Jace K Bauer, Ryan Nelson

**Affiliations:** 1 Orthopedic Surgery, A.T. Still University Kirksville College of Osteopathic Medicine, Kirksville, USA; 2 Orthopedic Surgery, Ascension Genesys Regional Medical Center, Grand Blanc, USA

**Keywords:** closed reduction of shoulder, hands-up posture, luxatio erecta, luxatio erecta humeri, inferior shoulder dislocation

## Abstract

Luxatio erecta humeri is an infrequent type of glenohumeral dislocation. The pathophysiologic mechanism responsible for luxatio erecta humeri is a traumatic incident of direct axial loading or a rapid hyperabduction injury. Patients commonly present with severe shoulder pain and the classic appearance of their arm externally rotated and stuck in abduction overhead. Radiographs help confirm the diagnosis by showing the humeral shaft parallel to the scapular spine and the humeral head inferior to the glenoid. Prompt recognition, neurovascular assessment, and reduction are necessary. This case details an incident of luxatio erecta humeri in an 83-year-old female after a ground-level fall with a history of ipsilateral rotator cuff repair greater than 20 years ago. She had subjective numbness in her fingers that resolved post-reduction. Anesthesia assisted in safely sedating the patient for orthopedics to successfully perform a closed reduction by combining techniques from the two most described maneuvers. The patient was discharged in a shoulder immobilizer for follow-up outpatient and later scheduled for reverse total shoulder arthroplasty.

## Introduction

Luxatio erecta humeri (LEH) is a very rare form of shoulder dislocation, accounting for only 0.5% of all glenohumeral dislocations, while anterior and posterior dislocations account for 95%-97% and 2%-5% of all shoulder dislocations, respectively [1-3]. A recent systemic review found only 199 total cases reported in the literature [4]. Such a low incidence may cause physicians to may misdiagnose LEH as an anterior or posterior dislocation and be unaware of the appropriate reduction maneuver, causing more harm to the patient. Of the reported cases in the literature 64% involved men, 44% were secondary to falls, 39% of cases involved proximal humerus fractures, and neurologic injury was reported in 29% of cases [4]. Though direct and indirect injury mechanisms exist to produce LEH, the indirect mechanism is most common [5,6]. A direct mechanism causes inferior glenohumeral dislocation when an abducted arm experiences a direct axial load, causing the humeral head to displace inferiorly through the weaker inferior glenohumeral ligament [3,5,7]. In this mechanism, shear forces between the humeral head and glenoid are generated, which commonly causes injuries to the glenoid labrum and humeral head, damaging the inferior glenohumeral ligament [8]. The indirect mechanism, from which LEH typically arises, involves a rapid hyperabduction of an already abducted arm [5,6]. This may occur when a patient attempts to break their fall by reaching and grasping onto something overhead. In this scenario, the acromion acts as a fulcrum onto the proximal humerus, causing the humeral head to dislocate from the glenoid cavity [5,6]. This indirect mechanism is more commonly associated with soft tissue injuries, especially to the middle and inferior glenohumeral ligaments, the inferior margin of the joint capsule, and the rotator cuff [5]. Due to the often-traumatic nature of this injury, secondary surveys become imperative. Understanding the patient’s baseline status and detailed histories aid in understanding the injury mechanism and goals of treatment.

LEH is more commonly reported in men and classically presents in a “hands-up” posture [1,8]. On physical exam, a large palpable mass, the humeral head, is found in the axilla with the arm stuck in abduction and external rotation. The forearm tends to be flexed and pronated, with the patient’s hand resting on or behind the head [5-8]. This is associated with severe pain and limited movement [9]. Radiographic imaging is diagnostic and classically portrays the humeral shaft parallel to the scapular spine with the humeral head inferior to the glenoid [4].

After performing a closed reduction, the shoulder should be immobilized for two to three weeks. Persistent pain and instability warrant further workup [7]. LEH is the most likely shoulder dislocation associated with neurovascular compromise. Typically, patients are treated nonoperatively with physical therapy and have full resolutions of complications within three years [3,4,9,10]. This case details an incident of LEH in an 83-year-old right-hand dominant female after a ground-level fall with a history of ipsilateral rotator cuff repair greater than 20 years ago.

## Case presentation

**Figure 1:**
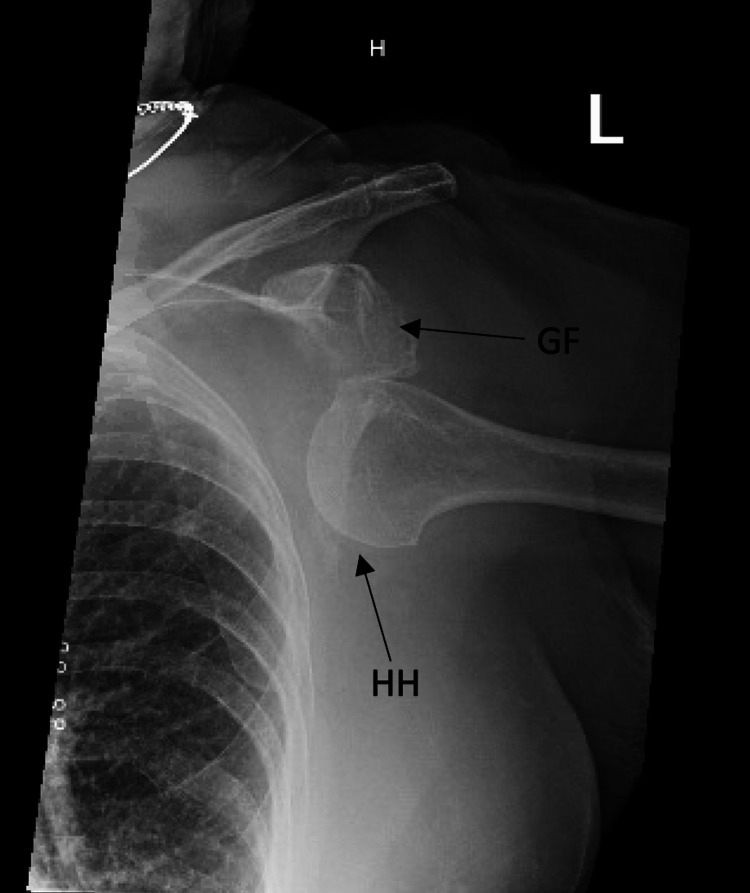
Anteroposterior view of the left shoulder demonstrating the humeral head inferior to the glenoid fossa. HH: humeral head, GF: glenoid fossa

**Figure 2:**
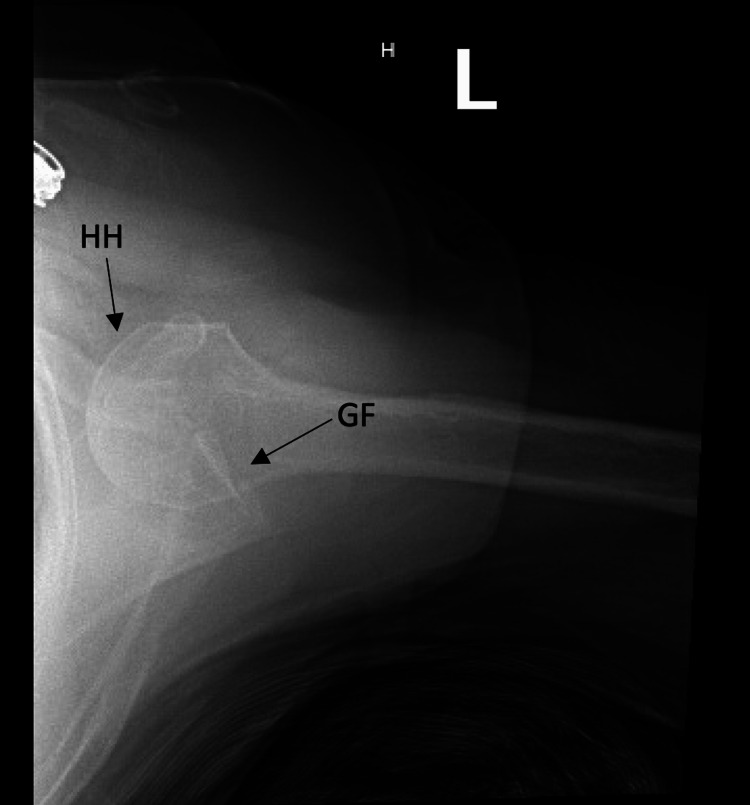
Axillary view of the left shoulder demonstrating inferior glenohumeral dislocation. HH: humeral head, GF: glenoid fossa

An 83-year-old right-hand dominant, self-ambulatory female with hypothyroidism, chronic obstructive pulmonary disease on two liters of home oxygen, chronic kidney disease stage 3, and a left shoulder rotator cuff repair 20 years prior arrived at the emergency department with a painful left shoulder fixed in abduction. While walking down a wheelchair ramp, the patient suffered a ground-level fall with her arm in forward flexion. She felt immediate shoulder pain and was unable to bring her arm down to her side from a fixed, abducted position. She was in acute distress, but alert and oriented to person, place, and time. She was hypertensive and tachypneic secondary to the left shoulder pain. The left arm was fixed in 120° of abduction, full external rotation, and 120° elbow flexion (her arm was above her head with her hand behind her head). The skin was intact. Radial and brachial pulses were intact and symmetrical to the contralateral limb, with a capillary refill time of less than three seconds. Aside from subjective numbness of all fingers, no brachial plexopathy or axillary nerve injury was seen. The secondary survey did not reveal any other injuries. Radiographs of the left humerus and shoulder demonstrated an anterior, inferior dislocation consistent with LEH (Figures 1, 2). No fractures were seen. The acromioclavicular joint was intact.

**Figure 3:**
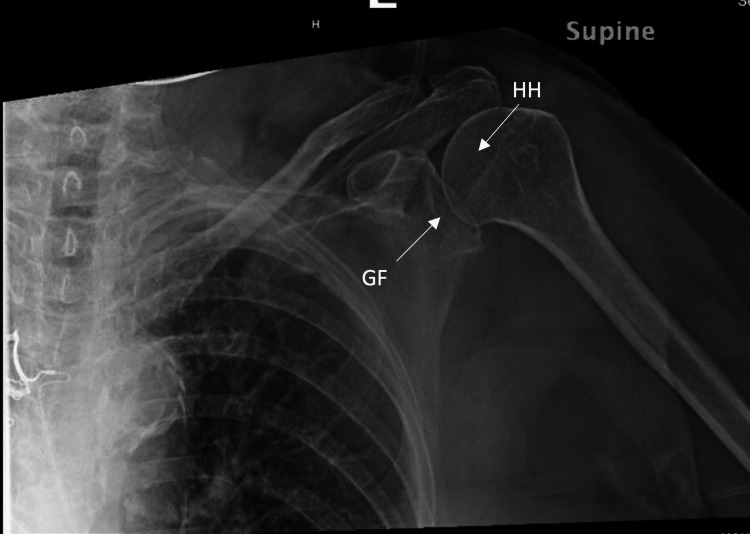
Anteroposterior view of the left shoulder postreduction demonstrating the humeral head within the glenoid fossa. HH: humeral head, GF: glenoid fossa

**Figure 4:**
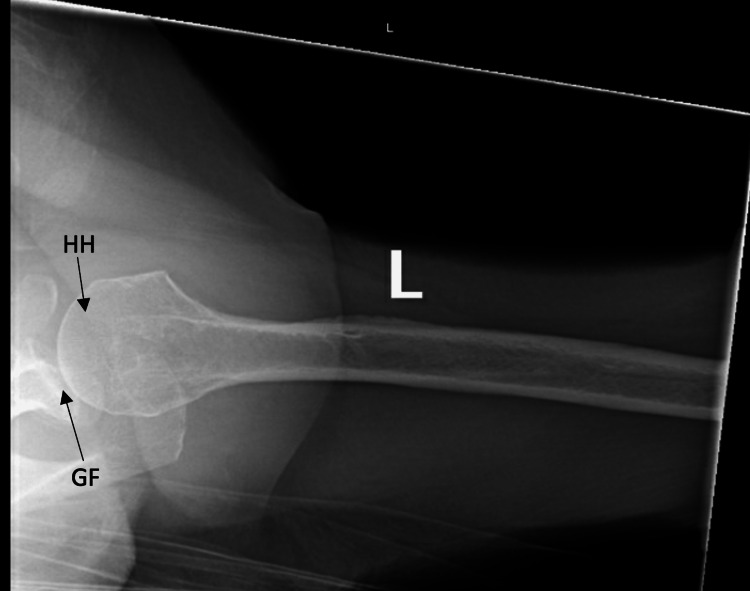
Axillary view of the left shoulder postreduction demonstrating a reduced humeral head within the glenoid fossa. HH: humeral head GF: glenoid fossa

Consent for closed reduction under conscious sedation was obtained. Then 15 mg IV etomidate was administered in 5 mg increments. Shoulder reduction was unable to be achieved secondary to inadequate muscle relaxation. Given concern for the patient’s medical comorbidities, anesthesia was requested to assist with bedside sedation. After 50 mcg fentanyl, 25 mg ketamine, and 20 mg propofol, the shoulder was safely reduced. Initially, a two-step maneuver was used, in which it was attempted to convert from inferior shoulder dislocation to an anterior shoulder dislocation. This was performed by pushing the lateral humerus and pulling at the medial epicondyle. Given how this technique was unsuccessful even with adequate and deeper sedation, a traction-counter-traction maneuver was utilized. Inline traction on the humerus was pulled superiorly with an assistant holding a rolled blanket over the shoulder providing countertraction in the opposite direction. In a sequential motion, the right hand grasped the arm just proximal to the elbow and pulled inline traction to the humerus superiorly. The left hand was in the axilla and pushed the humeral head from a posteroinferior position to an anterosuperior position. When a palpable clunk was felt the arm was brought from abduction and external rotation to adduction and internal rotation in a controlled, swift motion. Shortly after the first clunk, a second clunk was felt. This was likely the humeral head moving from an inferior dislocation to anterior dislocation to being reduced. Ultimately, the successful reduction occurred by using a combination of the two most described reduction maneuvers and altering the position of where the patient was grasped. After reduction, there was an ease of full shoulder range of motion with no feelings of instability. Post-reduction radiographs were ordered and showed a successfully reduced glenohumeral joint with no evidence of fracture (Figures 3, 4). The patient was then placed in a shoulder immobilizer with the arm in an adducted, internally rotated position.

**Figure 5:**
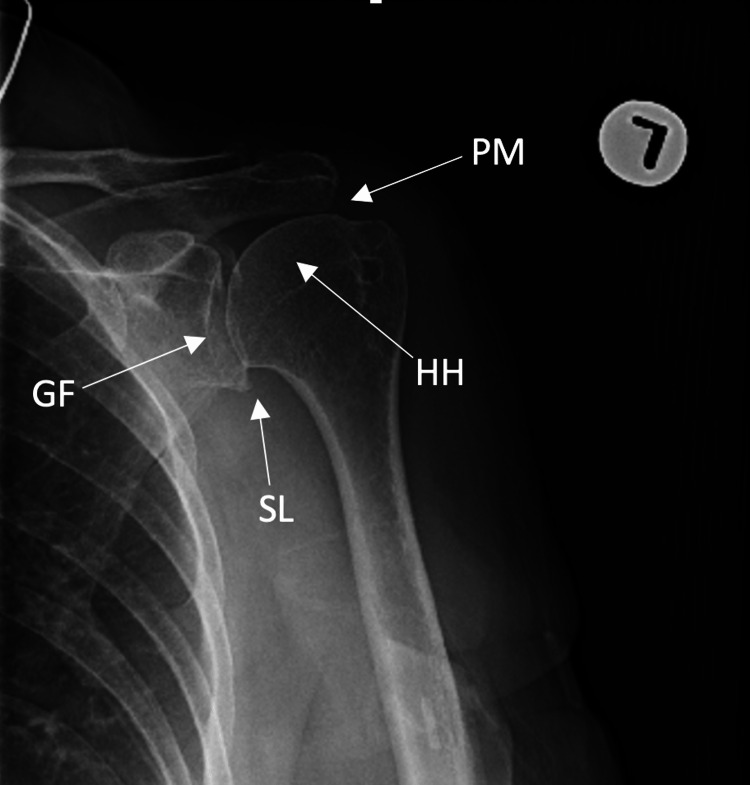
Anteroposterior view of the left shoulder demonstrating proximal migration of the humeral head, noted by disruption of Shenton’s line. HH: humeral head, GF: glenoid fossa, PM: proximal migration, SL: Shenton's line

**Figure 6:**
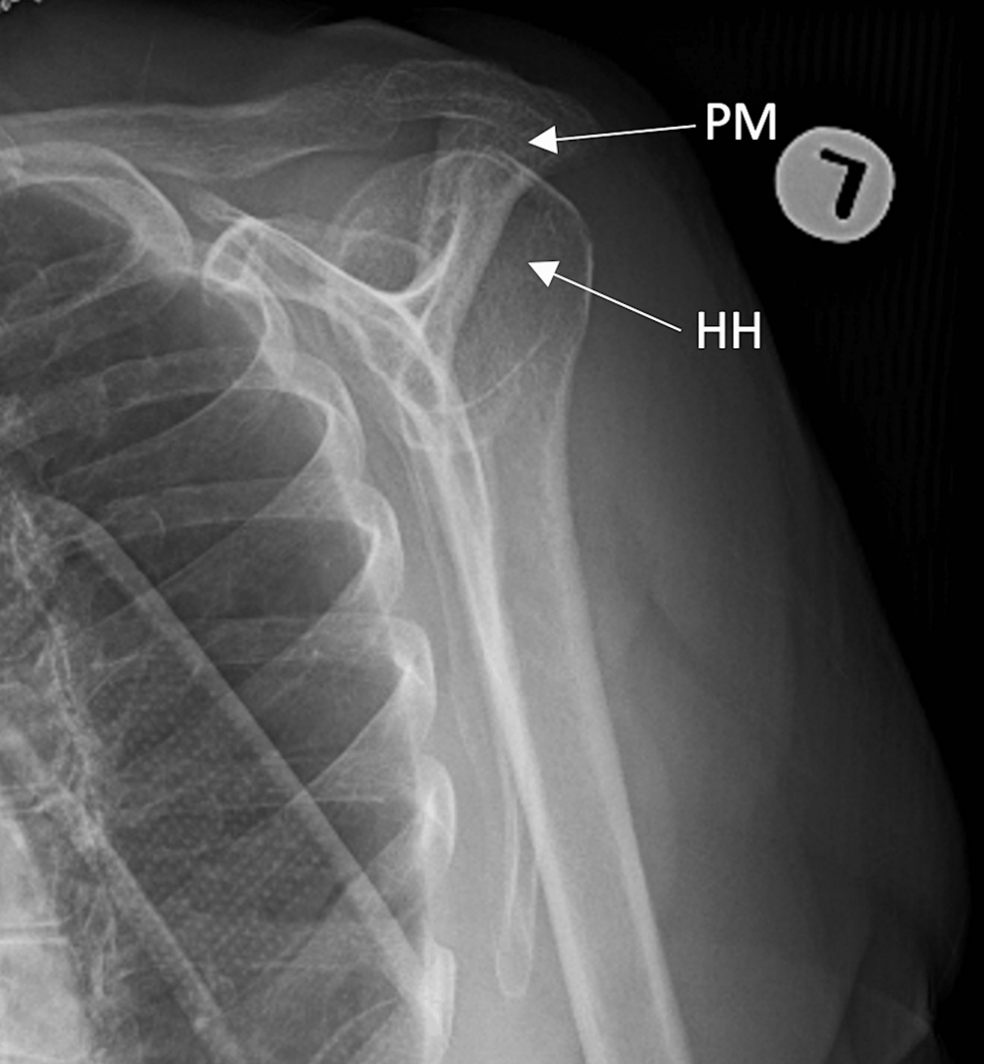
Scapular Y-view of left shoulder demonstrating humeral head within the glenoid with proximal migration. HH: humeral head, PM: proximal migration

The patient was discharged and instructed to maintain the shoulder immobilizer until follow-up outpatient with orthopedic surgery in 5-7 days. Pain medication was provided for the interim follow-up period. At her follow-up visit, radiographs showed the humeral head was located within the glenoid, but there was cephalad migration as noted by disruption to Shenton’s line, indicating signs of rotator cuff arthropathy (Figures 5, 6). Degenerative joint disease changes at the glenohumeral joint were noted. Clinically, this 83-year-old female, patient had evidence of rotator cuff re-tear in conjunction with functional deltoid. She had good bone stock. Therefore, at the time of writing, she was scheduled for reverse total shoulder arthroplasty.

## Discussion

LEH has an incidence of 0.5%, making it the rarest type of glenohumeral dislocation [1-10]. Inferior glenohumeral dislocations occur secondary to direct axial loads to an abducted arm or an indirect load caused by rapid hyperabduction to an abducted arm [5,6,8]. Prompt recognition, neurovascular assessment, and reduction are necessary to prevent complications. Diagnosis is made based on clinical and radiographic findings. Patients typically present with severe shoulder pain. Their arm is classically externally rotated and abducted with the forearm flexed and pronated, so the hand of the affected shoulder is resting on or behind the patient’s head, in a “hands-up” position [1,5,6,8]. Radiographically, this causes the characteristic findings of the humeral shaft parallel to the scapular spine and the humeral head located inferior to the lower rim of the glenoid fossa [4-6]. The humerus is held in an erect position by the pull of the pectoralis major muscle. The humeral head is palpable in the axilla. Posterior migration of the humeral head is counteracted by the long head of the triceps. The superior glenohumeral ligament prevents the humerus from dislocating anteriorly. The inferior forces on the humerus, keeping the humeral head in its position below the glenoid, are due to the pull of the teres major and latissimus dorsi [4,5]. Complete shoulder series radiographs are recommended to confirm the diagnosis of LEH, plan reduction maneuvers, and rule out any scapular or proximal humerus fractures [10].

There are two commonly described closed reduction techniques. The most common maneuver is an overhead traction-countertraction technique that requires a great deal of force and sedation to reduce the shoulder [8]. Another frequently used maneuver is gentler and requires less sedation since it first converts the inferior glenohumeral dislocation to an anterior dislocation before reducing the joint again [3]. If a reduction is unable to be achieved with sedation, the reduction maneuvers should be attempted under anesthesia [10]. After a successful closed reduction, conservative management with upper extremity immobilization for two to four weeks is the standard of treatment for glenohumeral dislocations, including LEH. Patients tend to recover well, especially with targeted physical therapy [1,2,9]. While neurovascular complications are most common with LEH compared to anterior or posterior shoulder dislocations, the neurovascular status is commonly restored postreduction [2,7]. If the vascular status is not restored, then surgical endovascular repair with open reduction is indicated [2,10]. Neurologic sequelae, if present, should be followed up appropriately and tend to resolve within a few years [9].

The most common musculoskeletal injuries associated with LEH are greater tuberosity fractures and rotator cuff tears [4,9]. Usually, a greater tuberosity avulsion fracture spares the rotator cuff from tearing. Other commonly associated injuries include labral tears, glenohumeral ligament tears, glenoid fractures, and humeral head fractures [7,9]. Impaction of the posterosuperior aspect of the humeral head onto the inferior glenoid rim can cause a Hill-Sachs-like lesion, which is more superior and lateral in location relative to the traditional Hill-Sachs lesion. This impaction is best evaluated by computed tomography [8]. A rarely reported long-term complication of LEH is adhesive capsulitis [10]. Operative treatment is indicated for concurrent rotator cuff tears or fractures of the proximal humerus [10].

Shoulder dislocations in the elderly and higher energy mechanisms, such as sports-related glenohumeral dislocations, are more likely to cause neurovascular compromise. Falls are a common mechanism of injury capable of producing LEH in the elderly because they land on a hyperabducted arm [2]. The patient presented here was an 83-year-old, with prior rotator cuff repair, placing her at a higher likelihood of neurovascular injury; however, only subjective numbness was noted at her fingers during the initial clinical exam, which later resolved post-reduction. Given her age and medical history, it was safe to have the anesthesia team at the bedside to assist in deeper sedation to help provide adequate muscle relaxation for successful reduction. Interestingly, the reduction maneuver that worked the best was ultimately a combination of the two most described techniques. Even though this patient had no neurovascular sequelae, it is important to recognize and manage this type of infrequent shoulder dislocation in a timely manner to avoid complications.

## Conclusions

LEH is the rarest type of shoulder dislocation commonly occurring in males and in trauma. Prompt recognition and reduction are required to avoid complications. Further reports of LEH will help elucidate the incidence of concurrent injuries and guide management principles. This report detailed a case of LEH in an elderly woman with prior ipsilateral rotator cuff repair. A combination of the two-step maneuver and traction-countertraction maneuver successfully reduced the glenohumeral joint. At her follow-up appointment, radiographs demonstrated a reduced humeral head with cephalad migration, degenerative changes, and disruption to Shenton’s line. Given these findings, she was scheduled for reverse total shoulder arthroplasty.
